# Cleroda-4(18),13-dien-15,16-olide as novel xanthine oxidase inhibitors: An integrated *in silico* and *in vitro* study

**DOI:** 10.1371/journal.pone.0253572

**Published:** 2021-06-30

**Authors:** Ha Thi Nguyen, Thien-Y Vu, Tikam Chand Dakal, Bhanupriya Dhabhai, Xuan Hong Quan Nguyen, Vinay Bharadwaj Tatipamula

**Affiliations:** 1 Institute of Research and Development, Duy Tan University, Da Nang, Vietnam; 2 Faculty of Medicine, Duy Tan University, Da Nang, Vietnam; 3 Faculty of Pharmacy, Ton Duc Thang University, Ho Chi Minh City, Vietnam; 4 Genome and Computational Biology Lab, Department of Biotechnology, Mohanlal Sukhadia University, Udaipur, Rajasthan, India; 5 Nguyen Thai Binh School, Ho Chi Minh City, Vietnam; Indian Institute of Technology Kharagpur, INDIA

## Abstract

In the present study, *in silico* predictions and molecular docking were performed on five clerodane diterpenes **(1–5)** from *Polyalthia longifolia* seeds to evaluate their potential as xanthine oxidase (XO) inhibitors. The initial screening was conducted by target prediction using TargetNet web server application and only compounds **3** and **4** showed a potential interaction with XO. Compounds 3 and 4 were subsequently subjected to *in silico* analyses on XO protein structure (PDB: 1N5X) using Schrödinger Release 2020–3 followed by structural modeling & molecular simulation studies to confirm the initial prediction result and identify the binding mode of these compounds to the XO. Molecular docking results revealed that compounds 3 (-37.3 kcal/mol) and 4 (-32.0 kcal/mol) binds more stably to XO than the reference drug allopurinol (-27.0 kcal/mol). Interestingly, two residues Glu 802 and Thr 1010 were observed as the two main H-bond binding sites for both tested compounds and the allopurinol. The center scaffold of allopurinol was positioned by some π-π stacking with Phe 914 and Phe 1009, while that of compounds **3** and **4** were supported by many hydrophobic interactions mainly with Leu 648, Phe 649, Phe 1013, and Leu 1014. Additionally, the docking simulation predicted that the inhibitory effect of compounds **3** and **4** was mediated by creating H-bond with particularly Glu 802, which is a key amino acid for XO enzyme inhibition. Altogether, *in vitro* studies showed that compounds **3** and **4** had better inhibitory capacity against XO enzyme with IC_50_ values significantly (*p* < 0.001) lower than that of allopurinol. In short, the present study identified cleroda-4(18),13-dien-15,16-olide as novel potential XO inhibitors, which can be potentially used for the treatment of gout.

## Introduction

Xanthine oxidase (XO) enzyme is abundantly expresses in the liver and intestine of the human body and plays critical roles in the last stages of purine metabolism [1]. Structurally, XO is a 290 kDa homodimer enzyme, of which, each subunit contains two spectroscopically distinct centers with one molybdopterin and one flavin adenine dinucleotide co-factor [2]. Biochemically, the molybdopterin center catalyzes the aerobic dehydrogenation of purine hypoxanthine to xanthine to uric acid and produces reactive oxygen species as byproducts [3]. Under normal physiological conditions, about 70% of the uric acid is excreted from the human body through the kidneys. Any conditions that lead to the excessive accumulation of uric acid inside the body, for instance, low excretion and/or over-production will cause hyperuricemia, which in turn, could lead to a type of painful inflammatory arthropathy commonly known as gout [4, 5]. The prevalence of gout varies across the world [6] and is estimated to occur in approximately 4.75% of European countries [6], 4% for USA [7], and >1% for Asia [6, 8] and Africa [6]. It has been shown that gout patients had higher risk for developing cancer, particularly cancer of the lungs, urological and digestive systems [9]. Besides this, during the catabolic process, a large amount of reactive oxygen species is generated, resulting in various oxidative stress complications such as diabetes [10]. Hence, controlling the uric acid levels by reducing the production of uric acid and/or increasing the excretion of uric acid from kidneys [11] is a promising approach to treat gout disease and reduces related complications.

XO inhibitor, allopurinol [1,5-dihydro-4*H*-pyrazolo [3,4-d]pyrimidin-4-one], is the commonly used drug for the clinical and therapeutic management of gout and its complications [12]. However, these drugs are contraindicated for a prolonged usage due to the known side-effects associated with extended use, including the development of skin rashes [13], renal failure [13], abnormalities in liver function [14], and hypertension [15]. Thus, there is great demand for alternative potent XO inhibitors from various natural sources with minimal or no adverse effects [2]. To date, numerous natural compounds including aloe-emodin analogs [16], curcumin [17], coumarins [18], chalcones [19], flavonoids [20], non-purine analogs [21], ellagic acid [22], naphthopyrans [23], hydroxychavicol analogs [24], valoneic acid dilactone [22], and polyphenols [25] have been identified and reported as XO inhibitors. However, none of these compounds have progressed to clinical trials due to a lack of adequate experimental evidence of drug-protein interactions. The interaction studies aid in understanding the binding mechanism and therapeutic potential of the potential drugs at the molecular level [26]. Recently, several studies have investigated the drug-protein interactions and binding mechanism of synthetic flavonoids to XO enzyme [2, 27, 28]. However, there are still no such studies on natural metabolites.

Recently, our group reported five clerodane diterpenes from *Polyalthia longifolia* (Sonn.) Thwaites, that were shown to have dual inhibitory properties against cyclooxygenases and lipoxygenases enzymes [29]. In the present study, we aimed to expand the study to predict the interactions of these clerodane diterpenes, to establish the compound-protein interactions by *in silico* studies and to investigate the *in vitro* inhibitory effects of these clerodane diterpenes against XO enzyme. The outcomes of this study are expected to provide valuable insights on the mechanism of action and therapeutic potential of these compounds and support the need for further clinical research on the use of clerodane diterpenes as XO inhibitors.

## Materials and methods

### Materials

**Fig 1:**
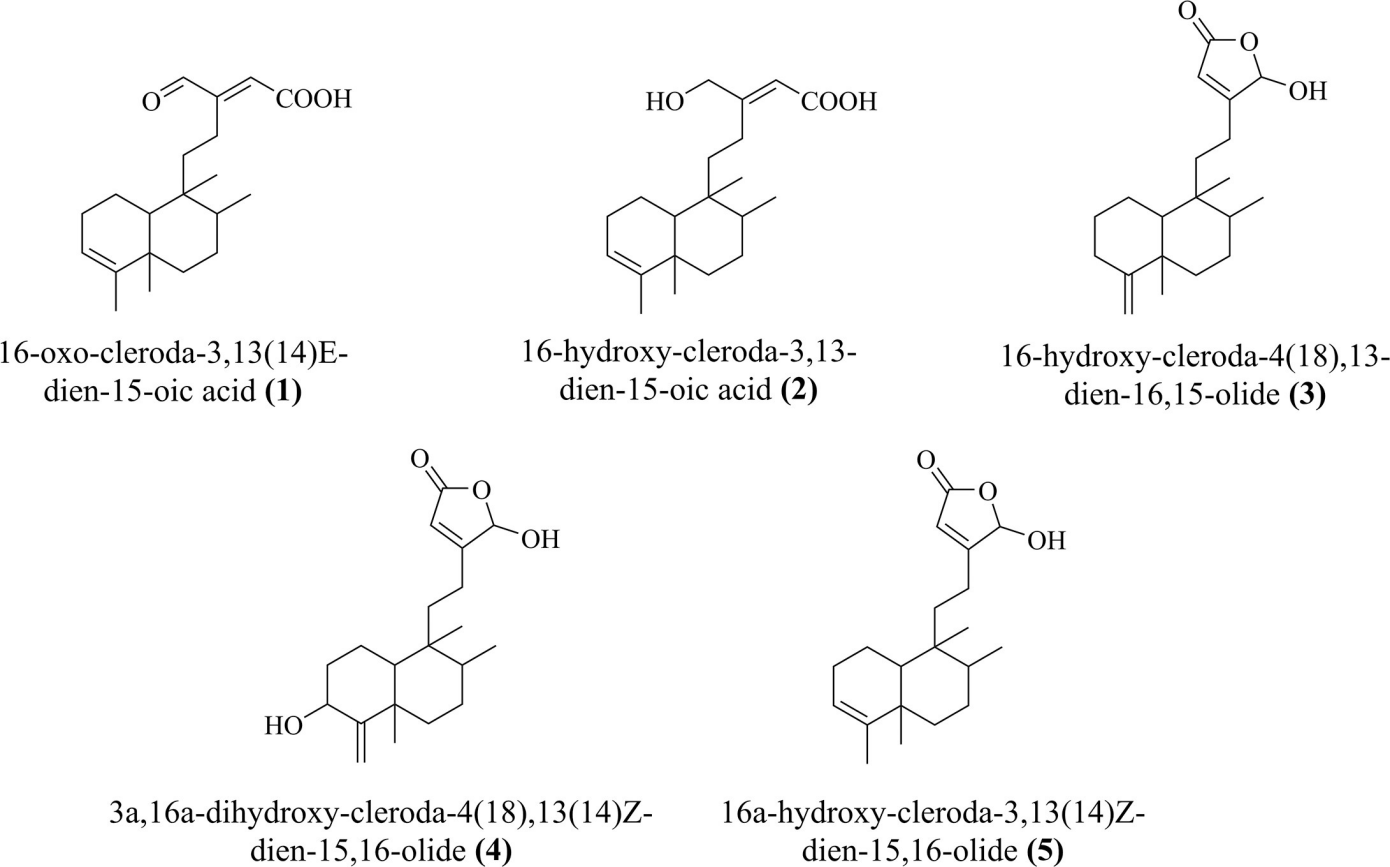
Chemical representation of clerodane diterpenes (1–5).

Five clerodane diterpenes (Fig 1) was previously isolated by our group from the methanol extract of *P*. *longifolia* seeds in a good yield [29].

### Prediction of five clerodane diterpenes’ targets

Target prediction of five cleodane diterpenes (**1**–**5**) was made using an integrative web application of TargetNet Server (targetnet.scbdd.com) [30, 31]. TargetNet server can make real-time potential target predictions based on input molecular structures. The compounds were input as canonical SMILE (simplified molecular-input line-entry) format and the output showed the potential targets having probability > 0.8.

### Compound docking and molecular dynamics simulations

The published crystal structure of XO (PDB: 1N5X) with Febuxostat drug was imported and prepared by the Protein Preparation Wizard [32] of Maestro software (Schrödinger Release 2020–3). Next, the structures of two clerodane diterpenes (**3** and **4**) and the standard drug allopurinol were generated and prepared by Ligprep [33] to attain different ionization states at biological pH (7.0 ± 2.0). The Standard-precision (SP), Extra-precision (XP) [34] docking and free binding energy estimations by molecular mechanics with generalized Born and surface area (MM-GBSA) method [35] were processed as previously described [29]. The experimental binding energies (Δ*G*_*exp*_) were approximately calculated from the measured inhibition concentrations (IC_50_ values) by using the equation Δ*G*_*exp*_ = *-RT*ln*IC*_*50*_, in which, the gas constant (R = 1.987 cal mol^-1^ K^-1^) and the temperature (T = 300 K).

Later, the system set-up of molecular dynamics simulations was constructed for a better understanding of the molecular mechanism of compound-protein interactions using Desmond [36]. The solvent model was set with flexible simple point-charge water model with OPLS3e force field. The total simulation time lasted 50 nanoseconds (ns) for each system and 50 picoseconds (ps) was predefined to trajectory recording intervals. At 1.01325 bar pressure and 300.0 K temperature, the ensemble class used was isothermal–isobaric ensemble and the system energy was 1.2. Before simulations, the relax model system was a default option.

### *In vitro* XO inhibitory assay

Two clerodane diterpenes (**3** and **4**) were subjected to XO inhibitory assay (Sigma Aldrich assay kit, Cat. No.: MAK078) as previously described [37]. Concisely, to 10 μl of the xanthine (substrate, 5 mM), added test sample (**3** and **4**) at four different concentrations (2.5, 5.0, 7.5, and 10.0 mg/ml), sodium phosphate buffer (470 μl, pH 8.5), and 10 μl of XO enzyme. The mixture was incubated at 25°C for 5 min and absorbance was measured at 295 nm against the blank (the test sample was replaced by methanol solvent). The percentage (%) of inhibition was calculated based on the absorbance values that in-turn was used to deliberate IC_50_ values using linear regression.

## Results and discussion

### Target prediction studies

By using TargetNet server, the clerodane diterpene’s targets were predicted based on the probability cut-off > 0.8. Among all, only compounds **3** and **4** showed to target xanthine dehydrogenase/oxidase (Table 1). These primary results provided a list of potential targets as well as potential biological activities of these compounds.

**Table 1:** Molecular targets of clerodane diterpene predicted using TargetNet.

Compound/compound	Protein	Probability
**1**	3-oxo-5-alpha-steroid 4-dehydrogenase 2	1
	Nitric oxide synthase, inducible	1
	Retinoic acid receptor RXR-beta	0.998
	Tyrosine-protein phosphatase non-receptor type 2	0.983
	Muscarinic acetylcholine receptor M4	0.921
**2**	Tyrosine-protein phosphatase non-receptor type 2	1
	Nitric oxide synthase, inducible	1
	Acetylcholinesterase	0.998
	3-oxo-5-alpha-steroid 4-dehydrogenase 2	0.996
	Arachidonate 15-lipoxygenase	0.994
**3**	Glutamate receptor ionotropic, NMDA 2B	1
	Estrogen receptor beta	1
	**Xanthine dehydrogenase/oxidase**	1
	Cathepsin L1	1
	Bifunctional epoxide hydrolase 2	1
**4**	Muscarinic acetylcholine receptor M2	1
	Beta-1 adrenergic receptor	1
	**Xanthine dehydrogenase/oxidase**	1
	Cathepsin L1	1
	Aromatase	1
**5**	Histone deacetylase 8	1
	Estrogen receptor	1
	Neprilysin	1
	Sodium channel protein type 5 subunit alpha	1
	Prostaglandin G/H synthase 2	1

### Docking studies

The superposition calculation of the native compound when docked onto XO recorded a root mean square deviation (RMSD) of 0.79 Å. This value showed a good binding mode of the Glide program, which was used for relative free binding energy MM-GBSA post-calculations. The results revealed that molecules **3 (**-37.3 kcal/mol) and **4** (-32.0 kcal/mol) have lower free binding energy as compared to the standard drug allopurinol (-27.0 kcal/mol). These values were consistent with the experimental data in which compound **3** had the best inhibitory ability against XO protein at -6.6 kcal/mol (Table 2).

**Table 2:** The XP docking and MM-GBSA values of two filtered compounds (3 and 4) and allopurinol.

Compounds	XO protein				
	XP GlideScore (kcal/mol)	MM-GBSA (kcal/mol)	Δ*G*_*exp*_ (kcal/mol)	No of H-bonds	Residues
**3**	-8.9	-37.3	-6.6	2	Glu 802, Thr 1010
**4**	-9.0	-32.0	-6.4	2	Glu 802, Thr 1010
**Allopurinol**	-5.9	-27.0	-6.2	2	Glu 802, Thr 1010

**Fig 2:**
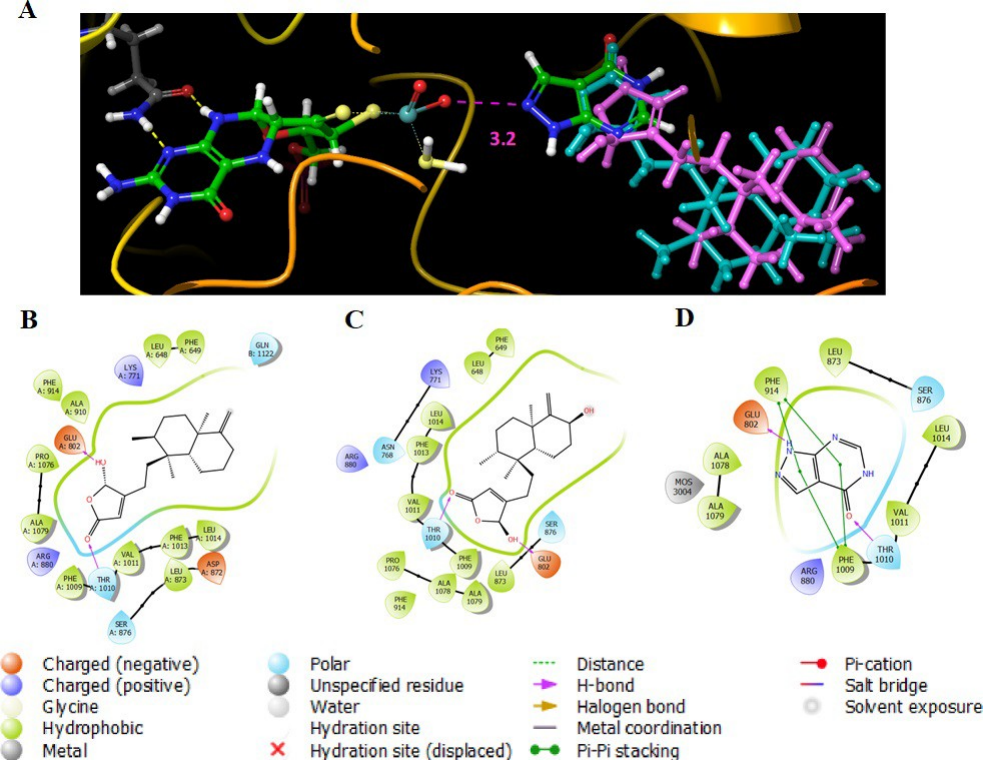
Docking interactions of compounds 3 and 4 against XO protein. (A) The distances between Mo complex and allopurinol (green and blue), compound 3 (mangenta), compound 4 (teal); Binding poses of (B) compound 3; (C) compound 4; and (D) allopurinol drug with XO protein. The additional informations in 2D diagrams present.

On the other hand, allopurinol had the shortest distance to the Mo complex at approximately 3 Å while that of compounds **3** and **4** were at ~5 Å (Fig 2A). The center scaffold of allopurinol drug was positioned by some π-π stacking with Phe 914 and Phe 1009 whereas compounds **3** and **4** were supported by many hydrophobic interactions with Leu 648, Phe 649, Phe 1013, Leu 1014, etc. However, two residues Glu 802 and Thr 1010 were observed as two main H-bond binding sites for all three of them (Table 2 and Fig 2B and 2C). These findings indicated that although there may be some differences in the binding mode, the main inhibitory activity of these two compounds is similar to that of allopurinol (Fig 2D). The detailed mechanism is further demonstrated in the section of molecular simulation below.

### Predicting the inhibitory mechanisms of compounds 3 and 4 by molecular dynamics simulations

**Fig 3:**
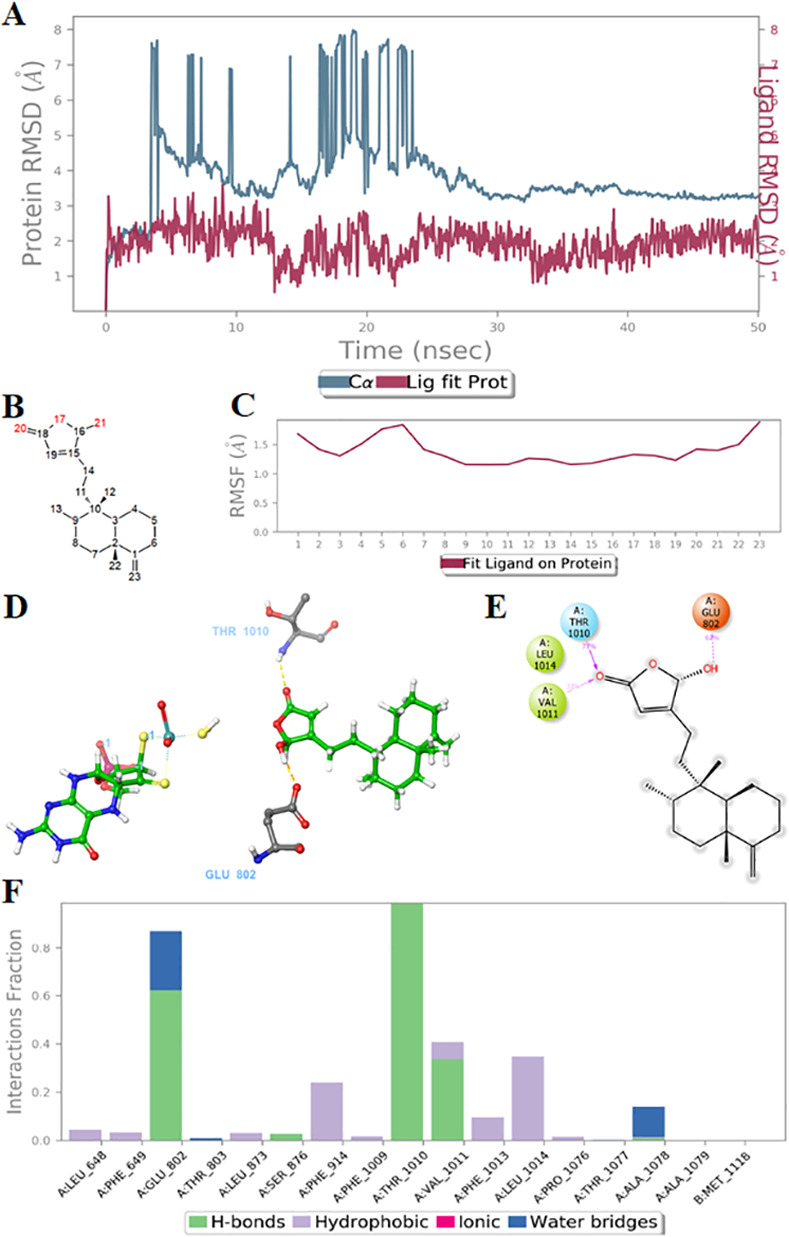
Molecular dynamics simulations of compound 3 –xanthine oxidase (XO) protein complex. (A) Root mean square deviation of protein (azure) and compound (red signal); (B). The number of atoms of compound **3**; (C) Root mean square fluctuation of compound **3** fitted on the XO protein; (D) 3D molecular dynamics simulations presenting binding modes of compound **3** to Glu 802 and Thr 1010 residues of XO *via* two H-bonds; (E) The common interactions (> 30.0% of the simulation time up to 50.05 nanoseconds); (F) The interaction percentage of compound **3** with surrounding residues. Green ball-and-stick: ligand; black: carbon atoms; red: oxygen atoms; blue: nitrogen atoms.

The molecular dynamic simulation was further implemented for compounds **3** and **4** with the simulation time of 50 ns. In compound **3**, the RMSD of ligand and protein was in the range 1–3 Å, signifying a perfect equilibrium of the system. Although, the RMSD of the protein fluctuated sharply at the early stages of the simulation, it stabilized at 25 ns and under 4 Å. On the other hand, the RMSD of the ligand was also regulated in the range of 3 Å, while the root mean square fluctuation (RMSF) of ligand fluctuated around 1.5 Å, indicating the tight binding of the ligand within the active site (Fig 3A–3C). Two residues Glu 802 and Thr 1010 were observed as the two main H-bond sites between the compound **3** and the protein. These specific interactions were maintained for 62 and 77% of the total simulation time, respectively (Fig 3D–3F and S1 Video). Glu 802 residue has been shown to be involved in the mechanism of action of XO enzyme inhibitory reaction [38, 39]. Particularly, a substitution mutation of Glu 802 by a Val has been proved to be associated with a reduction in XO activity paralleled with an 8-fold increase in *K*_*m*_ [40]_._ The Thr 1010 residue, on the other hand, was rarely mentioned in the previous studies. Its appearance could mainly be attributed to the structural suitability of the compound conformation [17].

Compounds **3** and **4** has great similarity in RMSD and RMSF. The additional hydroxyl group on the clerodane scaffold of compound **4** did not seem to alter the binding mode nor create any additional hydrogen bonds with backbone side chains as compared to compound **3** (S1 Fig). The H-bond with Glu 802 residue persisted at 100% total simulation time, whereas a H-bond could be formed directly or via water bridges between C = O oxygen of compound **4** with Arg 880 residue at 83% simulation time (S2 Video). The role of Arg 880 is equally important as that of Glu 802 because it stabilizes the charge buildup on C = O of the heterocyclic state during catalysis [39]. In short, these findings strongly indicated the key role of Glu 802 and Arg 880 residues in XO function, and compounds **3** and **4** conferred their inhibiting effect against XO enzyme by creating H-bond with these amino acids.

### *In vitro* XO inhibitory activity

**Fig 4:**
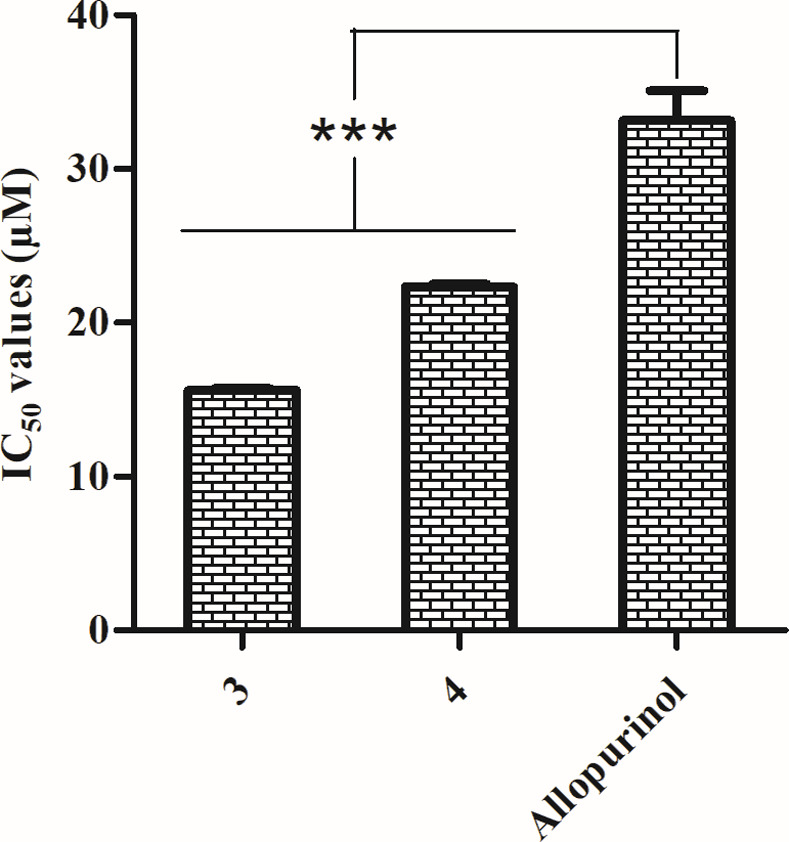
IC_50_ values of xanthine oxidase inhibitory activity of clerodane diterpenes (3 and 4). Values were presented as mean ± standard deviation (*n* = 3). Statistical analyses were performed using one-way ANOVA with Tukey’s multiple comparison test and *** means *p* < 0.0001.

The anti-XO effect of compounds **3** and **4** was evaluated based on their capability to inhibit the XO enzyme. The assay outcomes showed that clerodane diterpenes **3** and **4** have better (*p* < 0.0001) XO inhibitory activity as compared to the reference drug, allopurinol. The IC_50_ values of clerodane diterpenes **3** (15.63 ± 0.11 μM) and **4** (22.36 ± 0.15 μM) were significantly lower than that of the allopurinol (33.14 ± 1.96 μM) (Fig 4). Additionally, the cytotoxicity of these two compounds were performed on a human epithelial cell line and no toxicity was observed up to 10.0 mg/ml (S1 Table).

### Structure-activity relationship

**Fig 5:**
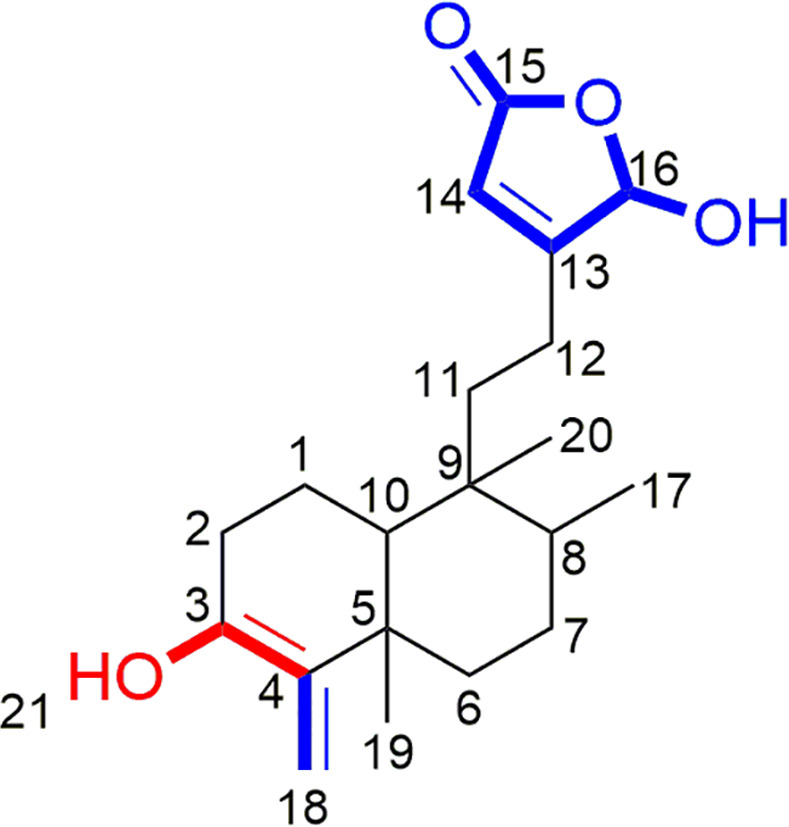
Structure-activity relationship of clerodane diterpenes as xanthine oxidase inhibitors. Red color (negative) and blue color (positive) indicate impacts on the biological activities of the clerodane diterpenes.

In the present study, the clerodane diterpenes (**3** and **4**) were identified to be very potent agents against XO. The results of biological assay and computational illustrations were in high concordance and both showed the inhibitory effects of compounds **3** and **4** on XO protein. Based on these results, the below structure-activity relationship could be determined for clerodane diterpenes: (i) The presence of 16-hydroxyfuran-15-one at C12 position and double bond (C = C) between C4 and C18 are crucial for the biological activity; (ii) Replacement of double bond (C = C) from C4-C18 to C3-C4 decreases XO inhibitory activity; (iii) The presence of hydroxyl group at C3 position also shortens the biological activity (Fig 5).

## Conclusions

To conclude, the present work was the first to demonstrate the inhibitory activity of clerodane diterpenes from *P*. *longifolia* seeds against XO. Initially, target prediction studies identified that only compounds **3** and **4** had binding interactions with XO. The subsequent docking study revealed that compounds **3** and **4** had great similarity in RMSD, RMSF and the interactions with XO protein. The molecular simulation studies revealed that both compounds **3** and **4** interacted with XO protein by creating H-bonds with Glu 802 residue. These findings were further supported by *in vitro* assay that showed more potent XO inhibitory activity of these compounds than that of the standard drug allopurinol. In short, the current study strongly indicated clerodane diterpenes as potent XO inhibitors that can be used in anti-gout drug development.

## Supporting information

S1 Fig Molecular dynamics simulations of compound 4 –XO protein complex. (DOC)

S1 Table Percentage of inhibition of compounds 3 and 4 on human epithelial cell line. (DOC)

S1 Video The MD simulations for ligand 3 and XO protein. (MPEG)

S2 Video The MD simulations for ligand 4 and XO protein. (MPEG)
