# Supplementary material for: Cleroda-4(18),13-dien-15,16-olide as novel xanthine oxidase inhibitors: An integrated in silico and in vitro study
Source: PLoS One. 2021 Jun 30;16(6):e0253572. doi: 10.1371/journal.pone.0253572 (PMC8244894; doi:10.1371/journal.pone.0253572)
Supplement: S1 Fig — (DOC) [file pone.0253572.s001.doc]

| **A** | 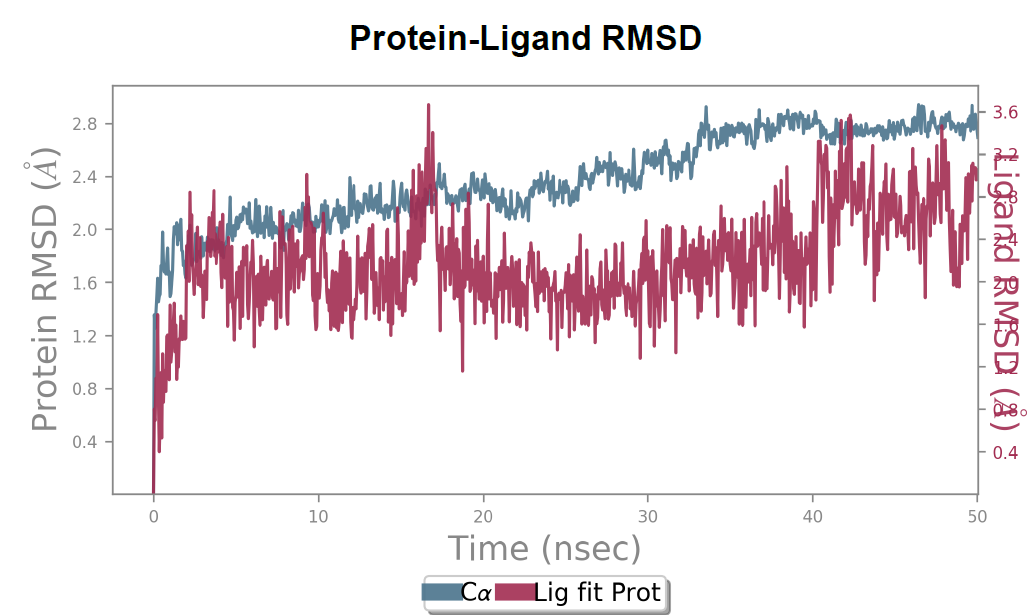 |
| --- | --- |
| **B** | **C** |
|  | 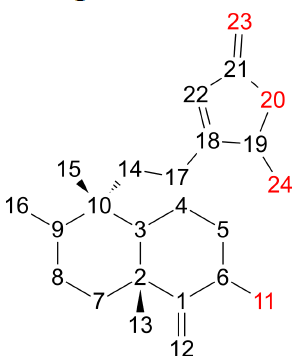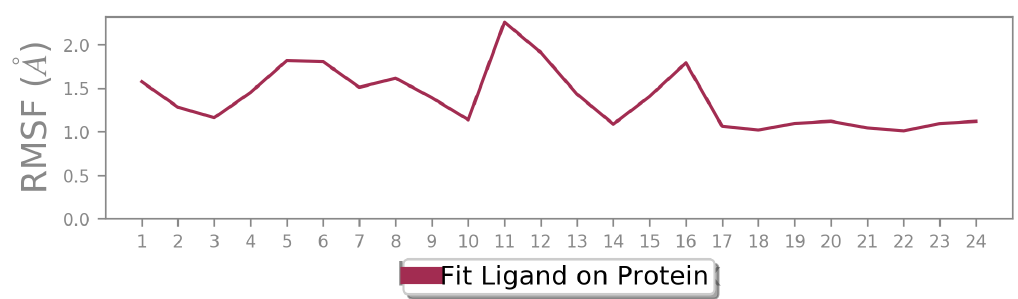 |
| **D** | **E** |
| **F** | 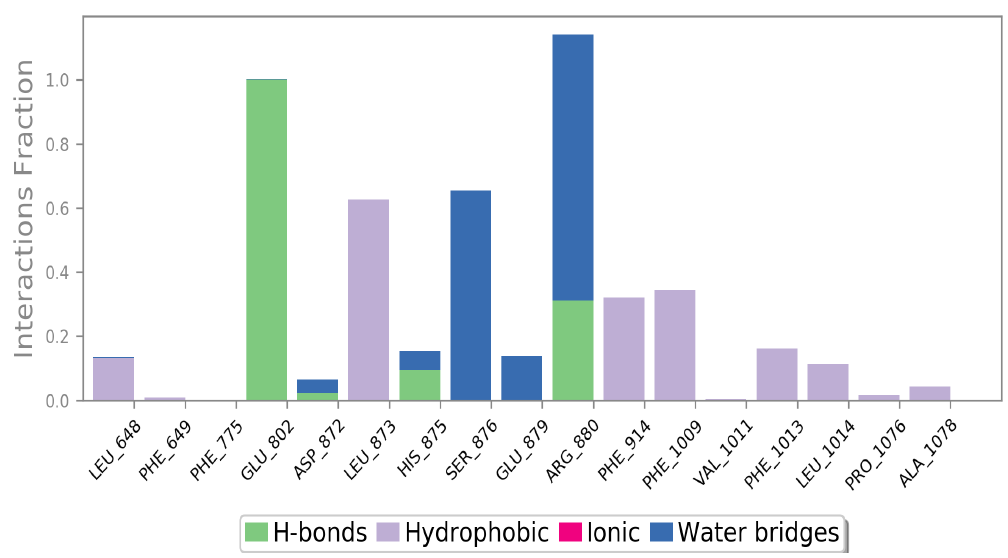 |

**S1 Fig. Molecular dynamics simulations of compound 4 – XO protein complex.** (A) RRMSD of protein (azure) and ligand (red signal); (B) The number of atoms of compound **4;** (C) RMSF of compound **4** fitted on the protein (red line); (D) Snapshots 3D from stable segments of molecular dynamics simulations show that compound **4** bound to Glu 802, Arg 880 residues by forming one H-bonds and one salt bridge; (E) The interactions that occur more than 30.0% of the simulation time in the selected trajectory (0.00 through 50.05 ns); (F) Interaction diagram demonstrates the percentage interaction of compound **4** with surrounding residues. Ligand is shown in green ball-and-stick, oxygen atoms in red, carbon atoms in green, nitrogen atoms in blue. XO: xanthine oxidase; RMSD: Root mean square deviation; RMSF: Root mean square fluctuation; ns: nanoseconds. No H-bond observed in the ligand – protein contacts of the additional hydroxyl group on clerodane scaffold of compound **4**.
