# Supplementary material for: Cleroda-4(18),13-dien-15,16-olide as novel xanthine oxidase inhibitors: An integrated in silico and in vitro study
Source: PLoS One. 2021 Jun 30;16(6):e0253572. doi: 10.1371/journal.pone.0253572 (PMC8244894; doi:10.1371/journal.pone.0253572)
Supplement: S1 Table — (DOC) [file pone.0253572.s002.doc]

**S1 Table. Percentage of inhibition of compounds 3 and 4 on human epithelial cell line**.

| **Compound** | **Percentage of inhibition (%) at different concentrations*** | | | |
| --- | --- | --- | --- | --- |
|  | **2.5 mg/mL** | **5.0 mg/mL** | **7.5 mg/mL** | **10.0 mg/mL** |
| **3** | 0.43 ± 0.18 | 1.38 ± 0.13 | 1.26 ± 0.16 | 2.18 ±0.16 |
| **4** | 1.43 ± 0.14 | 2.40 ± 0.69 | 3.21 ± 0.36 | 4.25 ± 0.28 |

*****mean ± standard deviation (*n* = 3)
